# Evaluation of ppd response in patients with idiopathic arthritis who are on biological drug therapy

**DOI:** 10.1186/1546-0096-12-S1-P6

**Published:** 2014-09-17

**Authors:** Kenan Barut, Muhammet Köşker, Tugba Erener-Ercan, Haluk Cokugras, Yildiz Camcioglu, Necla Akcakaya, Ozgur Kasapcopur

**Affiliations:** 1 Pediatric Rheumatology, Istanbul University, Cerrahpasa Medical Faculty, Istanbul, Turkey; 2 Pediatric Infectious Disease, Istanbul University, Cerrahpasa Medical Faculty, Istanbul, Turkey

## Introduction

Juvenile idiopathic arthritis (JIA) is a disease most commonly presenting as peripheral arthritis, and increased inflammatory response due to endogenous and exogenous antigens plays a role in its pathogenesis. Tuberculin skin test (TST) is used to determine whether a person is infected with Mycobacterium tuberculosis. In studies, delayed type hypersensitivity response was found to be suppressed in vitro among JIA patients. TST in JIA patients was studied for the first time by our group. In that study, PPD response was found not to be affected by the distribution of subgroups of the disease, activity of the disease or treatment (methotrexate, prednisolone). None of the enrolled children in that study were using biological drugs.

## Objectives

The aim of our study was to investigate the effect of biological drugs on PPD reaction mediated by Th1 immune response in a group of JIA patients who were not involved in our previous study.

## Methods

The study group consisted of 234 patients with the diagnosis of JIA according to ILAR diagnostic criteria and who were using biological drugs, and 45 healthy controls. PPD of the patients which was routinely done during the follow up was obtained from the patient files. BCG vaccination status of the patients and controls was similar. PPD values ≥ 5 mm were considered positive. Subjects with a PPD ≥ 10 mm were evaluated with the suspicion of tuberculosis infection and those with the final diagnosis of tuberculosis infection were given anti-tuberculosis drug therapy.

## Results

Of the 234 JIA patients on biological drug therapy**,** 102 (43.6%) were male and 132 (56.4%) were female. Age distribution was 3.25-19,8 years, mean age was 12.8 ± 4.7 years. Disease duration ranged between 3 months and 17 years and mean duration of disease was 5.9 ± 4.11 years. Mean diameter of PPD indurations’ was 4,996± 6,8494 mm (diameter: 0- 40 mm) in JIA patients and 7.83 ± 3.47 mm (diameter 0–16 mm ) in controls (p = 0,0056). PPD positivity was found in 96 (%41) and 38 (%84,4) of the subjects in JIA and control groups, respectively **(p<0,0001).** PPD≥10 mm was detected in 59 (%25,2) and 19 (%42,2) subjects of the JIA and control groups, respectively **(p=0,03).** PPD was negative in 125 (%53,4) and 3 (%6,6) of the subjects in JIA and control groups, respectively **(p< 0,0001).** When PPD induration was reported with respect to the biological drugs that were used; PPD was 4.632±6.3490 mm in 185 (79,1%) of the subjects using etanercept (p=0,01), 4.957±9.1873 mm in 23 (9,8%) of the subjects using adalimumab (p=0,06), 8.391±7.7386 mm in 23 (9,8%) of the subjects using infliximab (p=0,6) and 1.667 ± 2.8868 mm in 3 (1,3%) of the subjects using anakinra. There was no difference in PPD response between patients using etanercept and adalimumab. PPD induration was larger in subjects using Infliximab.

## Conclusion

In patients with JIA who had infantile BCG vaccination and who were currently on biological drugs, PPD induration was significantly lower compared to the control group. There was no difference in PPD response between patients who were on biological drugs or who were not using drugs according to our two major studies. Prospective studies evaluating measurements of PPD reaction in patients on biological drug therapy would provide further information on this issue.

## Disclosure of interest

None declared.

